# An Evaluation of the Clinical Assessments of Under-Five Febrile Children Presenting to Primary Health Facilities in Rural Ghana

**DOI:** 10.1371/journal.pone.0028944

**Published:** 2011-12-13

**Authors:** Frank Baiden, Seth Owusu-Agyei, Justina Bawah, Jane Bruce, Mathilda Tivura, Rupert Delmini, Stephaney Gyaase, Seeba Amenga-Etego, Daniel Chandramohan, Jayne Webster

**Affiliations:** 1 Malaria Group, Kintampo Health Research Centre, Kintampo, Ghana; 2 Department of Disease Control, London School of Hygiene and Tropical Medicine, London, United Kingdom; Walter & Eliza Hall Institute, Australia

## Abstract

**Background:**

The shift to test-based management of malaria represents an important departure from established practice under the Integrated Management of Childhood Illnesses (IMCI). The possibility of false results of tests for malaria and co-morbidity, however, make it important that guidelines in IMCI case assessment are still followed.

**Methods and Findings:**

We conducted a cross-sectional observational study to evaluate current practices in IMCI-based assessment of febrile children in 10 health centres and 5 district hospitals, with follow up of a subset of children to determine day 7–10 post-treatment clinical outcome. Clinical consultation, examination and prescribing practices were recorded using a checklist by trained non-medical observers. The facility case management of 1,983 under-five years old febrile children was observed and 593 followed up at home on days 5–10. The mean number of tasks performed from the 11 tasks expected to be done by the IMCI guidelines was 6 (SD 1.6). More than 6 tasks were performed in only 35% of children and this varied substantially between health facilities (range 3–85%). All 11 tasks were performed in only 1% of children. The most commonly performed tasks were temperature measurement (91%) and weighing (88%). Respiratory rate was checked in only 4% of children presenting with cough or difficulty in breathing. The likelihood of performing “better than average number of tasks” (>6) was higher when the consultation was done by medical assistants than doctors (O.R. = 3.16, 1.02–9.20). The number of tasks performed during assessment did not, however, influence clinical outcome (O.R. = 1.02, 0.83–1.24).

**Conclusion:**

Facility-tailored interventions are needed to improve adherence to IMCI guidelines incorporating test-based management of malaria. Studies are needed to re-evaluate the continued validity of tasks defined in IMCI case assessment guidelines.

## Introduction

Millennium Development Goal four (MDG-4) is to reduce the number of under-five year old deaths from 93 to 31 per 1000 by 2015. Prompt and appropriate management of febrile illnesses in under-five years old children is one of the interventions that would contribute to the achievement of MDG-4 [1], [2]. Currently the management of fevers in low-resource settings is based on the WHO and UNICEF-supported Integrated Management of Childhood Illnesses (IMCI). The IMCI guidelines aim to reduce childhood mortality and disability due to common illnesses, and improve growth and development. It also aims at improving health-worker performance, strengthening health systems, and enhancing family and community practices [3], [4], [5], [6].

Prior to the introduction of IMCI, guidelines for the management of childhood illnesses were disease-specific [7], [8]. The major justifications for introducing IMCI were: (1) pneumonia, diarrhoea, malaria, measles and malnutrition accounted for over 70% of child deaths in many settings; (2) high frequency of co-morbidities (malnutrition is known to underlie 40–60% of other illnesses); (3) considerable overlap in clinical presentations of several common childhood illnesses; (4) first-level facilities generally lack laboratory capacity and practitioners rely only on clinical signs and symptoms to manage childhood illnesses [9], [10], [11].

Under IMCI, the case management of a sick child is expected to follow a stepwise approach that includes proper assessment, classification, treatment and counselling [3], [12], [13]. The assessment step involves checking vital signs, identifying danger signs, and enquiring about the presence of four main symptoms. It also includes enquiring about other problems and assessing the nutritional and immunization status of the child [12]. IMCI has recommended that in high malaria risk areas (where more than 5% of the fever cases in children are due to malaria), any child who presented with fever should be given an ant malarial [12], [14].

The Ministry of Health in Ghana adopted the IMCI approach in 1998 and by 2000 it was rolled out in all primary care facilities within the Ghana Health Service (GHS) [15]. The case management guidelines have been adapted and incorporated into the curricula of health worker training programmes in the country.

The presumptive approach to managing febrile illness has contributed to the over-diagnosis of malaria and the excessive use of antimalarials, including the relatively expensive artemisinin-based combination treatments (ACTs) [16], [17]. There is now however a shift to test-based management of malaria. The successful application of this shift in under-five children inevitably requires that it is incorporated into IMCI, the standard approach to managing childhood illnesses. WHO has accordingly reviewed the IMCI guidelines in order to incorporate test-based management of malaria as a requirement in endemic countries. For health systems and clinicians who have for many years followed the presumptive treatment approach of IMCI, adhering to the new guidelines will be a challenge [18]. In order to understand the potential barriers in introducing the new IMCI guidelines, we investigated current IMCI-based assessments of sick children in primary care facilities in rural Ghana. The study was part of a larger study to evaluate the health system determinants of RDT-based ACT use in rural Ghana.

## Methods

### Study area and selection of facilities

The study was conducted in six out of the 19 districts in the Brong Ahafo Region of Ghana. The study districts (Kintampo North, Kintampo South, Nkoranza North and South, Tain and Techiman districts) lie within the forest-savannah transition belt of the country and are largely rural. Malaria transmission is year round in the study area and is the leading cause of out-patient attendance. The entomological inoculation rate is estimated at 269 infective bites per person per year [19].

The districts were selected due to their longstanding working relationship with the Kintampo Health Research Centre. Five district hospitals were purposively selected and ten health centres were randomly selected using probability proportionate to size, based on 2008 under-five OPD attendance in all health centres in the five districts. One district hospital was excluded because a study that would compete with our study was running in the facility. No prior study in IMCI or intervention in clinical case management had been conducted in any of the sampled facilities. Test-based management of malaria had not been introduced into routine care in any of the facilities at the time of the study.

### Study design and procedures

A cross-sectional survey was conducted between May 2009 and October 2009 in the 15 selected health facilities. The survey included non-participant structured observations of the management of febrile children, exit interviews with carers of the sick child, health worker interviews and health facility audits. The non-participant structured observations were used to assess the application of IMCI guidelines for management of febrile children. This was conducted in out-patient departments of the study facilities during normal working hours (0800–1600 hrs), from Monday to Friday. Carers of children aged 3 months to 59 months presenting with fever or a history of fever were approached and informed of the study. Research staff approached the carer of the first eligible child they came across at the OPD, obtained written informed consent, and observed the encounter between health workers, the child, and the child's caretaker. Observations were recorded on a structured checklist adapted from the generic instrument used in the IMCI multi-country evaluation [20], with additional notes where appropriate. The observation checklist included actions taken by the health workers, and verbal communication between the health workers and carer from the point of registration through history taking and consultation through to the point of exit. After completing the observation of first child they selected the next eligible child they could find. No staff observed two children at the same time.

Data collectors had a minimum of a high school certificate, at least three years of research experience and were eligible for engagement in the GHS as trainee technical officers. They undertook a three-week training program that included introduction to IMCI guidelines and routine activities in health facilities, and training in conducting non-participant structured observations. The program consisted of eight days of didactic sessions and 10 days of practice at the Kintampo Municipal Hospital. After pre-testing and adapting the data collections forms, inter-research staff variability was assessed to ensure a minimum of 95% concordance among the data collectors on the parameters of IMCI assessment when the same child is observed.

Meetings were held with the authorities and health workers of the facilities to explain the objectives of the study and to reassure that the research staff would not interfere with the work of the health workers, but rather would observe and complete the checklist.

In five facilities (2 health centres and 3 hospitals) that were selected for logistical feasibility of follow-up, all eligible children attending the facility over a period were followed-up at home, 7–10 days after presentation, to assess the outcome of case management.

Ten per cent of the observations were re-checked by a research officers for quality control by comparing the data recorded in the observation checklist with health facility records.

### Sample size

The sample size estimation was based on the primary outcome of the larger study which was designed to estimate the frequency of a range of steps in the delivery of effective treatment with an ACT for malaria diagnosed according to IMCI guidelines. An estimated 556 febrile children were needed to measure outcomes that occur with a frequency of 50% with a 95% confidence level of +/− 5% and a design effect of 2 to account for potential clustering within health facilities. The required sample size to maintain this 5% precision at the endpoint of delivery based upon hypothetical estimates of children lost from the evaluable sample at each step of delivery; 60% diagnosed with malaria, 70% prescribed ACT, 90% given correct dose, was 1,471 children. Based upon the final sample size for the structured observations of 1,983 (see results section), this study has the power to detect an outcome with 50% frequency with a 95% confidence interval of +/−3% and a design effect of 2.

### Data management and analysis

Data were double-entered and validated using customised data entry screens in FoxPro version 6. Analyses were conducted using Stata version 10 (StataCorp, College Station, Texas).

A list of 11 tasks required to be performed (according to the GHS-adapted WHO-UNICEF IMCI case management chart) was used to develop an index to assess health worker performance [21]. These tasks were the assessment of danger signs (ability to drink and breastfeed, vomits everything, history of convulsion or convulsing at presentation, lethargy or unconsciousness), the four main symptoms (fever, cough and difficulty in breathing, diarrhoea and ear problem) and other general measures of health (malnutrition or anaemia, immunization and other problems).

Descriptive analysis of key provider, health system, carer and child factors was performed. The arithmetic mean of the number of IMCI assessment tasks performed was computed. The mean number of tasks performed was 6 (SD 1.6) and thus the level of adherence to IMCI guidelines was deemed to be “*better than average*” if the number of tasks performed were ≥7. Univariate analysis was used to identify factors associated with *better than average* performance. All analysis were adjusted for clustering in health facilities. The extent of heterogeneity (design effect) between health facilities for the *better-than-average* performance and its component variables was assessed. The consistency in data collectors grading was assessed by examining the association between individual data collectors and level of adherence to IMCI assessment, after adjusting for variation across health facilities.

### Ethical approval

The study was approved by the ethics review committees of the GHS, the Kintampo Health Research Centre and the London School of Hygiene and Tropical and Hygiene (LSHTM). Administrative approval was obtained from the respective district and hospital management teams, while health workers gave written consent to have the consultations and other procedures observed by a third party. Individual informed consent was obtained from all carers.

## Results

The catchment population of the studied health centres ranged from 1,789 to 47,025, while that for hospitals ranged from 5,214 to 202,409 (Table 1). The majority of facilities were government-owned, and headed by non-physicians. Malaria smear microscopy was available in three health centres and in all five district hospitals. While in 4 facilities, no staff had had training in IMCI, in 7 other facilities, only one staff had received such training at the time of the study. The average OPD-staff to catchment population ratio was 1: 5,768 for health centres and 1: 6,774 for the hospitals.

**Table 1:** Characteristics of the participating facilities and assessment of IMCI performance.

FACILITY	Nature of facility	Catchment population	No of staff	No of staff who manage OPD for sick children	No of staff in OPD trained in IMCI	Under-5 malaria cases in preceding year	Number of children observed	Percentage of children receiving *better-than-average* assessment (n)
Health centre 1	Government	4844	3	1	1	840	75	85 (64)
Health centre 2	Government	12594	5	4	1	3402	75	72 (54)
Health centre 3	Government	17219	4	4	0	5995	77	49 (38)
Health centre 4	Government	47025	9	4	1	4286	75	35 (26)
Health centre 5	Government	14568	5	2	0	3032	75	32 (24)
Health centre 6	Government	1789	9	4	1	3913	76	30 (23)
Health centre 7	Government	20335	5	4	1	7193	78	13 (10)
Health centre 8	Government	15358	5	3	1	1972	75	12 (9)
Health centre 9	Government	39941	8	3	1	8371	179	9 (16)
Health centre 10	Private	4765	2	2	0	5730	76	3 (2)
Hospital 1	Mission	202409	168	39	9	9036	200	58 (115)
Hospital 2	Government	72676	71	11	11	1287	359	46 (164)
Hospital 3	Mission	137726	71	7	6	26284	260	36 (92)
Hospital 4	Government	24412	23	18	2	2833	152	32 (48)
Hospital 5	Government	5214	10	5	0	4014	151	14 (21)

A total of 1983 structured observations were undertaken over a period of six months; 1122 in the health centres and 861 in the hospitals. Follow-up of children to assess health outcomes on day 7–10 post-management started later than the main study. This delay resulted in additional structured observations and therefore an increase in the overall number of observations conducted.

The mean age of the children observed was 21months (3–59 months), with 19.1% aged less than one year. There were slightly more males (53%) than females. The majority (88%) of children were brought in by their mothers, of whom 6% were teenagers. Approximately one third of carers had no formal education.

The most frequent time of registration for children was between the hours of 1000 hrs and 1200 hrs, with 45% of children registering between these hours. About 90% of children were registered with the national health insurance scheme. The sequence of activities followed by the majority (89%) of children was registration at the out-patient department, history-taking and vital signs assessment, consultation, collection of drugs, followed by exit from the facility. Only 11% of children were referred to the laboratory for investigations and 1% referred to higher level facilities for further assessment.

Common presenting symptoms besides fever (which was an inclusion criterion) were cough (33%), vomiting (30%), diarrhoea (20%) and loss of appetite (25%). Consistently, symptoms were reported more often during consultation than during history-taking and vital signs assessments. Temperature and weight were checked in 91% and 88% of children respectively. In only 4% of children presenting with cough or difficulty in breathing was respiratory rate measured.

The proportion of children assessed for each task in the national IMCI guidelines are shown in Table 2. Among the danger signs the assessment of ability to drink (21%) and history of convulsions (13%) was very low. Only 15% of children were assessed for ear problems and the immunisation status was checked in 20% of children.

**Table 2:** IMCI-based parameters and number of children in which each parameter was assessed (N = 1983).

Parameter	Number (%) of children assessed
**Danger Signs**	
Ability to drink and breastfeed	406 (21%)
Extent of vomiting	1066 (54%)
History of convulsions or convulsing at presentation	259 (13%)
Lethargy or unconscious	1970 (98%)
**Main Symptoms**	
Presence of fever	1983 (100%)
Cough and difficulty in breathing	1338 (68%)
Diarrhoea	1352 (68%)
Ear problem	305 (15%)
**Others**	
Malnutrition & anaemia*	1913 (97%)
Immunization	391 (20%)
Other problems	817 (41%)

*Based on child weighing and assessment of pallor.

All 11 items in the IMCI checklist were assessed in only 1% (11) of children and all of these children were observed in the same facility. *Better-than-average* (≥7 required tasks performed) IMCI assessment was achieved in 706 (35%) children with considerable variation (3% to 85%) across the facilities (Table 1). The worst performance (3%) was recorded in the only private facility among the facilities studied. The estimated design effect for the primary outcome measure of *better-than-average* IMCI assessment was 23.9.

Whereas nearly all (95%) children received an antimalarial, only 30% received an antibiotic. Twenty-nine percent of those who received an antibiotic received an antimalarial in addition.

### Outcome of case management

Among the 593 children followed up 7–10 days after they were seen at the health facility, 560 (94%) were reported to have “improved” by their carers. The condition of 30 (5%) was reported as “unchanged”. Caretakers of 3 children reported a “new illness” or worsening of the existing condition. Four (0.7%) children were lost to follow-up. The outcome of case management did not differ according to the extent of IMCI assessment (O.R.1.02, 0.83–1.24).

### Predictors of *better-than-average* assessment

Compared with doctors, *better-than-average* assessment was likely to be achieved when a medical assistant conducted consultation. Although nurses appeared to perform better than doctors, the difference was not statistically significant. Similarly, the likelihood of *better-than-average* assessment did not differ according to whether a nurse or health assistant performed history-taking and took vital signs (Table 3).

**Table 3:** Association between various factors and a better-than-average IMCI assessment (N = 1983).

			EXTENT OF IMCI ASSESSMENT		O.R.	CI	P-value
			better-than-average	Average or lower			
**Provider factors**	**Cadre of staff doing history & taking vital signs**	Nurse	538	902	1.32	0.65–2.70	0.42
		Health assistant	168	372			
	**Cadre of staff consulting**	Medical assistant	439	514	3.16	1.02–9.20	0.05
		Nurse	110	194	2.03	0.69–5.97	
		Non-professionals	6	113	0.19	0.03–1.21	
		Doctor	76	272			
**Health System Factors**	**Type of facility**	Health centre	440	682	1.44	0.53–3.94	0.44
		Hospital	266	595			
	**Child health insurance status**	Insured	608	1168	0.57	0.24–1.36	0.19
		Uninsured	98	108			
	**Mode of registration**	Paid money	39	23	3.19	0.63–16.22	0.15
		Free	667	1253			
	**Time of registration**	Before 12	487	882	1.00	0.71–1.40	0.98
		After 12	219	395			
	**OPD-staff to population ratio (among health centre)**	Less than 5768	200	332	2.40	0.49–11.66	0.24
		More than 5768	66	263			
	**OPD-staff to population ratio (among hospitals)**	Less than 6774	348	514	1.24	0.52–2.92	0.53
		More than 6774	92	168			
	**Time spent in history-taking and vital signs assessments**	More than 3 mins	174	283	1.33	0.83–2.11	0.50
		2–3 mins	174	251	1.44	0.88–2.35	
		1–2 mins	130	204	1.28	0.83–1.96	
		Less than 1 min	223	464			
	**Time spent in clinical consultation**	More than 8 mins	179	264	1.39	0.84–2.30	0.35
		4–8 mins	289	529	1.12	0.72–1.75	
		Less than 4 mins	233	478			
	**Time spent at the facility**	More than 90 mins	184	306	1.30	0.58–2.91	0.47
		60–90 mins	165	209	1.71	0.71–4.12	
		30–60 mins	199	373	1.15	0.58–2.30	
		Less than 30 mins	136	294			
	**Attendance at the facility on the day child reported**	Higher than average	321	570	0.97	0.71–1.31	0.81
		Lower than average	205	352			
	**Having at least one IMCI-trained staff**	Yes	621	983	2.19	0.71–6.70	0.16
		No	85	294			
**Carer factors**	**Sex of carer**	Female	669	1214	0.94	0.55–1.59	0.80
		Male	37	63			
	**Age of carer**	<20 yr	39	86	0.87	0.62–1.21	0.38
		≥20 yr	548	1048			
	**Ability to speak any one of the three major dialects**	Yes	74	110	1.44	0.69–3.00	0.30
		No	632	1181			
	**Carer's educational level**	Secondary Education	51	102	0.83	0.45–1.53	0.80
		Primary	426	794	0.89	0.55–1.44	
		None	229	381			
	**Relation with child**	Mother	617	1126	0.92	0.67–1.28	0.61
		Other	89	150			
**Child factors and presenting symptoms**	**Age of child**	≤12 mths	47	71	1.21	0.65–2.26	0.52
		>12 mths	659	1206			
	**Sex of child**	Male	368	688	0.93	0.81–1.06	0.25
		Female	337	585			
	**Cough**	Yes	265	384	1.40	0.92–2.12	0.11
		No	441	907			
	**Vomiting**	Yes	225	373	1.13	0.86–1.48	0.36
		No	481	918			
	**Diarrhoea**	Yes	229	342	1.13	1.04–1.67	0.03
		No	477	949			
	**Abdominal Pain**	Yes	120	240	0.88	0.64–1.22	0.43
		No	586	1051			
	**Other**	Yes	249	524	0.78	0.60–1.03	0.07
		No	457	767			

Adjusted for clustering within health facilities.

Achieving *better-than-average* assessment did not depend on whether a child was assessed at a health centre or hospital (OR = 0.69; 0.26–1.89), or whether the child was covered under the NHIS or not. The amount of time spent in history taking, vital signs, and clinical consultation had no significant effect on the extent of performance. (Table 3) The overall time spent at the facility was not a predictor of performance. The extent of assessment was similarly not significantly influenced by whether the child attended the clinic on a day when attendance was higher than the average facility attendance (0.97; 0.71–1.31) or whether the facility had at least one staff trained in IMCI (2.19; 0.71–6.70).

None of the carer-related factors explored had a significant effect on the extent of assessment (Table 3). Among the children-related factors however, the likelihood of *better-than-average* assessment was slightly higher in children who presented with a history of diarrhoea, compared to children without diarrhoea (1.13; 1.04–1.67).

There were no differences between data collectors in their IMCI assessments, adjusted for the facility that each of them conducted observations in (P = 0.16).

## Discussion

The study findings apply directly to the facilities evaluated, and are representative of health centres in the 6 districts studied. Because the study was undertaken in a period just before the introduction of test-based management of malaria, findings may serve as baseline against which to assess the impact of test-based management of malaria on IMCI assessments in the future.

The Hawthorne Effect is an acknowledged limitation of non-participant structured observation, the key method used in this study [22], [23], [24]. Health workers who are conscious of the fact they were being observed may have altered their behaviour. We assume in this study that if the health workers behaviour was changed because they were being observed then it was changed in a positive direction so that what was observed was the health workers improved practices. In our analysis, we used the mean assessment across all children in all facilities to define a cut-off of IMCI assessment. Our choice of this cut-off does not represent an endorsement as either satisfactory or adequate, the mean number of tasks performed by the health workers. This cut-off was taken in the absence of any standard in the literature on the assessment of IMCI case management practices. This approach to analysing health worker performance, and the tendency of health workers towards improved practice increased the potential for high scores in the assessment of their performance. In spite of this, the extent of execution of the required tasks was very low. In only 1% of children were all 11 required tasks performed.

The findings of this study are consistent with the evaluations of IMCI case management practices in many other similar settings [13], [], [25], [26,27]. Inadequate health-worker performance is a very widespread problem [28]. It is perhaps reasonable to begin to question whether, at the current level of health worker motivation and the overburdened heath systems, the standards defined in IMCI-based case management are implementable. There is a need for qualitative studies to assess health worker perceptions of the usefulness of the various tasks included in IMCI-based case management, and the practical barriers to their implementation. The pattern of assessments found in this study is similar to that observed in Benin where fever was among the most assessed and ear problems among the least assessed [25]. Complaints of diarrhoea elicited *better-than-average* IMCI assessment suggesting that clinicians intuitively considered whether there was the need to assess all parameters in each case. This could result in overlooking one of the objectives of the IMCI case management process, which is to use clinic visits by sick children, irrespective of the type of illness, as an opportunity to deliver preventive interventions [29].

Our finding that clinical outcome was not significantly influenced by the extent of IMCI assessment raises questions about the efficacy of the tasks listed within IMCI guidelines. Ninety-four percent of children had a favourable outcome and this was irrespective of the extent of IMCI assessment. However, in our study we did not have expert re-evaluation of the study children to compare the treatment decision that would have been made if the IMCI guidelines had been strictly adhered to. Thus the lack of an association between the level of assessment of IMCI indicators and the health outcomes observed in this study should be interpreted with caution. Our observation is nevertheless consistent with another study in Benin where the children were re-evaluated by an expert clinician, it was found that although health workers often deviated from IMCI guidelines, it did not necessarily lead to ineffective treatment [25].

We were unable to assess IMCI performance matched against individual health worker attributes including whether IMCI trained or otherwise. However the presence of at least one staff member who had received training in IMCI at the facility did not make any difference to the assessments conducted at the facility. Although training and re-training has often been proposed as an intervention to improve health worker performance, its impact remains unclear. While some reports suggest a positive impact of training [25], [26], [29], others report no effect [30], [31].

After 15 years of IMCI implementation and little evidence of its effective implementation or effectiveness (possibly due to poor implementation), a re-evaluation of its guidelines, algorithms and approach is required. Alongside re-evaluation of the tools for IMCI assessments, studies of its feasibility of implementation under a variety of health systems contexts, and assessment of factors that increase feasibility of effective implementation should be studied. Any revision to the IMCI guidelines should be undertaken in consultation with stakeholders, including clinicians and other health workers who are the users of the guidelines. A convincing evidence-base should be established to make the case for the inclusion or exclusion of each of the tasks on the IMCI assessment checklist.

Respiratory infections are an important cause of febrile illnesses in children in sub-Saharan Africa. In the absence of x-ray and laboratory support, IMCI case management guidelines stress the need to use respiratory rate in assessing and classifying acute infections. This is to provide a rational basis for the use of antibiotics and other interventions [11], [12], [32], [33]. Therefore our finding that respiratory rate is rarely measured in the assessment of febrile children suggests that the use of antibiotics in the management of acute respiratory infections is, in many cases, not based on rigorous clinical assessment.

In the process of assessing febrile children in health facilities in Ghana, there are two stages at which thorough history-taking and assessment is possible. These are during vital signs assessment and during consultation. Our finding that symptoms tend to be reported more frequently during consultation than during vital signs assessment suggest a need to strengthen the stage of history-taking and vital signs assessments. A probable solution may be the use of structured symptoms and signs forms that make it a requirement to record the findings of specific assessments at each stage. The use of such a form will offer clinicians and other health personnel a tool for self-circumspection. It will also provide real-time objective assessment of the extent of completeness of IMCI-based assessment procedures and a trail for the audit of case management procedures.

It has been argued that the management of non-malarial febrile illnesses will be improved by confirming the diagnosis of malaria [15], [17], [34]. However because of the possibility of false results and co-morbidity, using rapid tests to confirm malaria would still require clinicians to conduct thorough assessment in arriving at a definitive or working diagnosis [10], [35]. In the era of test-based management of malaria, safeguards need to be instituted to prevent the substitution of thorough clinical assessment with sole reliance on rapid test for malaria to classify febrile illnesses. That path would appear effortless and in the face of dwindling health staff and increasing workload on a few, there is danger that overreliance on rapid test for malaria could result in a further slip in the quality of clinical assessments. Our finding that antibiotics were nearly always given with an antimalarial (but not vice versa) suggests a lack of confidence with the diagnosis of non-malarial illnesses, together with wasteful use of antimalarials.

A striking feature in the results of this study is the lack of statistically significant associations between nearly all the factors that were investigated as potential predictors of the extent of IMCI assessment. However, the ORs showed trends towards association that may have been statistically significant given a larger sample size. We assume the lack of statistically significant associations was due to the loss of power as result of the large design effect of 23.6, for which adjustment was made in the analysis. Although typically, a design effect of 1–3 is anticipated and adjusted for in cluster surveys it is not uncommon for the design effect to be much larger [36], [37]. This level of design effect was not anticipated at the time of designing the study. However had such a large effect been anticipated, it would have been logistically impossible to conduct a study of a sufficient sample size to achieve a power of 80% in estimating the parameters of *better-than-average* IMCI assessment. The lesson in this regard is similar to that reported in the multi-country evaluation of IMCI [38]. We believe future studies that explore the quality of IMCI assessment across health facilities should consider the potential for a high design effect to undermine the power to detect significant associations and explore alternative approaches. The large design effect also points to considerable heterogeneity across health facilities in IMCI assessments and makes the case for facility-tailored interventions.

Our finding that most of the history-taking and vital signs assessments were conducted by health assistants, and medical assistants (in hospitals), and nurses and other cadres of staff conducted clinical consultations is a pointer to the extent that task-shifting is being employed to overcome the shortage of health workers in these facilities. This observation is consistent with reports from other African countries where task-shifting has been implemented [39], [40], [41]. Note should however be taken of the significantly poorer performance by non-professionals during clinical consultation. By existing GHS regulations, staff such as health assistants are not expected to conduct consultations and are therefore not targeted in IMCI training programs. The finding requires further investigation as it may be highlighting the limits to the extent that task-shifting can be taken in the delivery of specialised services.

### Conclusion

We have found adherence to national IMCI case management guidelines to be rather poor and below expectations, with practices varying widely across different facilities. However this did not appear to influence clinical outcome. We believe that facility-tailored interventions are needed to improve IMCI case assessments, and that the continued validity of tasks defined in IMCI case assessment guidelines need to be re-evaluated.

## Acknowledgments

We are grateful to the parents and guardians who permitted the assessments of their children to be observed in the various health facilities. We are similarly grateful to the heads and staff at the various facilities used for data collection, and the respective District and Hospital Management Teams. We wish to acknowledge the contribution of staff who undertook data collection, management and statistical analyses. We are particularly grateful to Mr. George Adjei. Our sincere thanks also go to the KHRC Scientific Review Committee, the ethics committees (KHRC, GHS LSHTM), project DSMB and the ACT Consortium.
